# The Isthmian Canal Problem

**Published:** 1905-04

**Authors:** 


A Monthly Review of Medicine and Surgery.

*EDITOR:*

WILLIAM WARREN POTTER, M. D.

All communications, whether of a literary or business nature, books for review and exchanges, should be addressed to the editor 284 Franklin St., Buffalo, N. Y.

Vol. XLIV.—LX. APRIL, 1905. No. 9

### The Isthmian Canal Problem.

EVERYONE at all familiar with the course of tropical diseases, especially as related to previous work upon the Isthmian Canal, knows that the underlying problem of its successful completion is the adequate sanitation of what is known as the canal zone, including also the cities of Panama and Colon.

Fully appreciating this fact the administration at Washington practically directed the canal commission to place at the head of the sanitary corps Colonel William C. Gorgas, assistant surgeon-general, United States army, who had previously dealt with the yellow fever problem at Havana, in a manner which entirely wiped out a pestilence that had propagated itself for over three hundred years. In spite of the fact, however, that Colonel Gorgas and his efficient staff, consisting of Major La Garde, Lieutenant Lyster, Dr. Carter and others,—subordinates of the highest type of efficiency, trusted lieutenants whose usefulness was known and appreciated,—were believed to be on duty in the field of canal operations, yellow fever appeared and began its deadly work.

It is hardly necessary to say that this circumstance surprised the people, especially those familiar with the work of Colonel Gorgas and his sanitary experts in the republic of Cuba. The mystery, however, appears to be solved by the report of Dr. Charles A. L. Reed, of Cincinnati, the more important parts of which are published elsewhere in this issue. In this document it is shown that Colonel Gorgas took timely action with reference to the prevention of both yellow fever and malarial disease, but it appears, also, that his efforts were thwarted by conditions over which he had no control and for which he was not responsible.

Dr. Reed's report was published in full in the *Journal of the American Medical Association* of date March 11, 1905, from which we make extensive excerpts, printed elsewhere under the head of Topics of Public Interest, leaving out only a few comparatively unimportant matters of detail. It will afford interesting reading for physicians who wish to keep pace with sanitary conditions relating to the construction of the canal.

It is true the Isthmian canal commission has made answer to this paper in a communication addressed to the Secretary of War, but in the published excerpts that we have seen adequate answer to Dr. Reed's criticisms does not appear. The President and the Secretary of War, under letters dated March 17 and 20, respectively, censure Dr. Reed for the premature publication of his report in a medical journal,—that is to say, because it was so published without the permission of the Secretary of War and before the commission, itself, had an opportunity to reply. However this may be, and we have no disposition to enter into a discussion of the official proprieties governing such questions, it still remains that, up to the present writing, the public has not been told why yellow fever and malarial disease were permitted to gain a footing in that part of the Isthmus over which the government exercises sanitary control.

The Japanese have taught the world a valuable lesson in military sanitation and hygiene. Medical officers precede the troops on the march, inspecting water and food supplies, habitations and all sources of possible infection, posting notices and guards to warn the approaching columns of every probable danger; and in camps or cantonments the greatest sanitary circumspection is exercised. Their small sick list and low death rate testify to the skilfulness and perfection of their sanitary administration.

The Isthmus of Panama presents problems similiar to those involved in a military campaign, the only difference being in the permanency of occupation; for, whereas, in war the armies frequently change position, the canal population on the contrary will remain in the allotted zone for an indefinite term of years. This increases the responsibility, makes it more weighty, and demands the exercise of all the skill and executive capacity belonging to the most superior and advanced sanitary science. Without a clean bill of health, so far as preventable diseases are concerned, the canal can never be constructed; and this is what makes the responsibility of our government one of awful proportions. We have all confidence that it will rise to the highest plane of exalted duty. We could wish that Leonard Wood, William C. Gorgas, and John Findley Wallace might be commissioned to finish the canal.

